# Genome Sequence of a Yunnan Orbivirus Isolated from a Dead Florida White-Tailed Deer (Odocoileus virginianus)

**DOI:** 10.1128/MRA.00168-21

**Published:** 2021-05-06

**Authors:** Pedro H. O. Viadanna, Thaís C. S. Rodrigues, Kuttichantran Subramaniam, Juan M. Campos Krauer, John A. Lednicky, Julia C. Loeb, Samantha M. Wisely, Thomas B. Waltzek

**Affiliations:** a Department of Infectious Diseases and Immunology, College of Veterinary Medicine, University of Florida, Gainesville, Florida, USA; b Emerging Pathogens Institute, University of Florida, Gainesville, Florida, USA; c Department of Large Animal Clinical Sciences, College of Veterinary Medicine, University of Florida, Gainesville, Florida, USA; d Department of Wildlife Ecology and Conservation, University of Florida, Gainesville, Florida, USA; e Department of Environmental and Global Health, College of Public Health and Health Professions, University of Florida, Gainesville, Florida, USA; KU Leuven

## Abstract

We report the complete coding sequences of a Yunnan orbivirus isolated from a dead white-tailed deer (Odocoileus virginianus) in Florida in 2019. The prevalence of Yunnan orbivirus and its role in disease among farmed white-tailed deer remain to be determined.

## Abstract (precis)

We report the complete coding sequences of a Yunnan orbivirus isolated from a dead white-tailed deer (Odocoileus virginianus) in Florida in 2019. The prevalence of Yunnan orbivirus and its role in disease among farmed white-tailed deer remains to be determined.

## ANNOUNCEMENT

Orbiviruses (family *Reoviridae*) possess genomes composed of 10 double-stranded RNA (dsRNA) segments that encode structural (VP) and nonstructural (NS) viral proteins (1, 2). They are transmitted to mammals by hematophagous arthropods, including *Culicoides* midges (3), mosquitoes (4), phlebotomine sand flies, and ticks (1, 2). Pathogenic orbiviruses, as well as other orbiviruses of unknown pathogenicity, have recently been isolated from farmed white-tailed deer in Florida (5–9).

**FIG 1:**
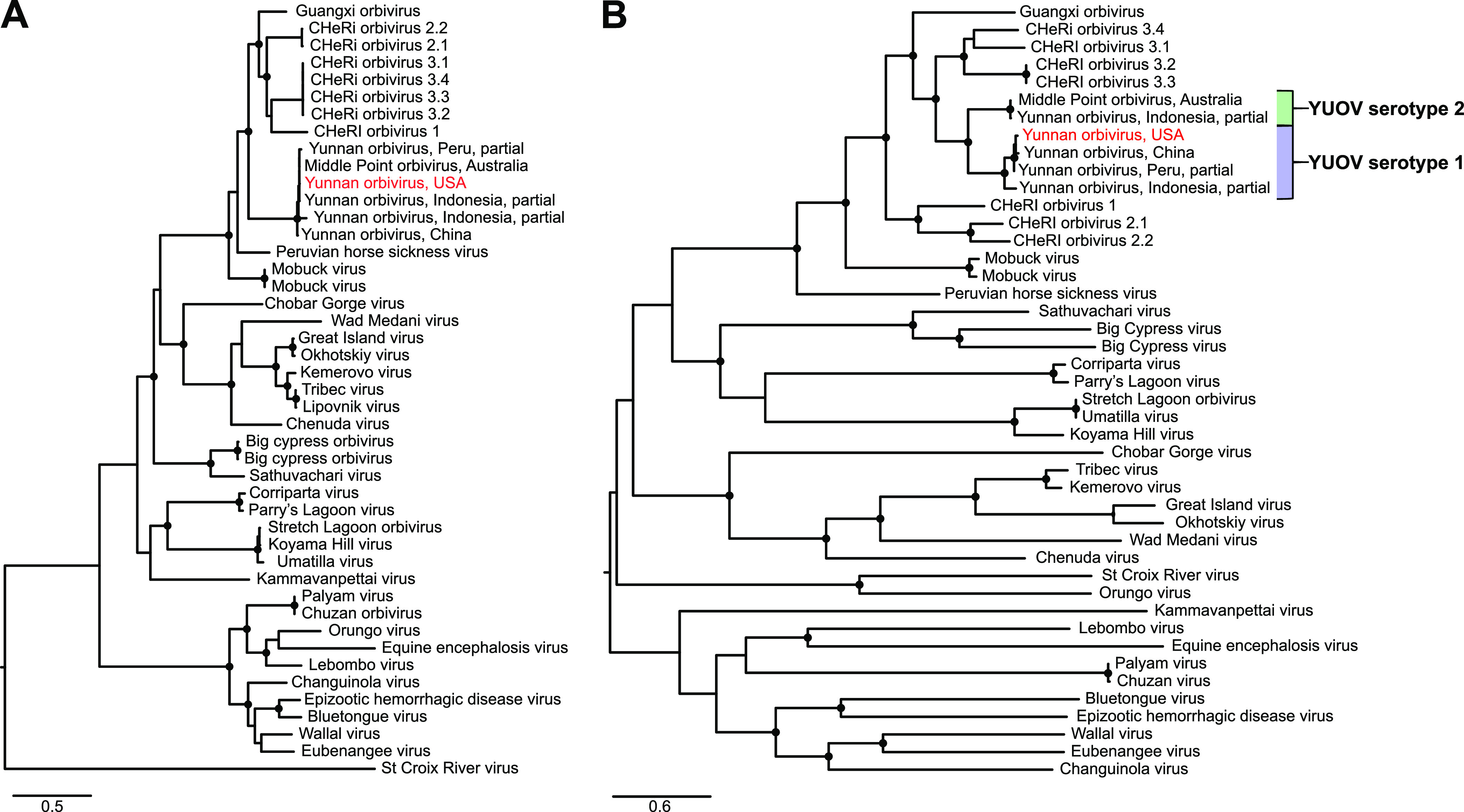
Maximum-likelihood phylograms depicting the relationship of the Yunnan orbivirus isolated from a white-tailed deer (OV1288) to other orbiviruses based on the amino acid sequence alignments of the VP2 proteins (A) and the outer capsid proteins (VP3) (B). YUOV OV1288 is highlighted in red. Serotype 1 YUOVs are indicated by a blue bracket, and serotype 2 YUOVs are indicated by a green bracket. All nodes with black circles are supported by bootstrap values of >90%. The branch lengths represent the number of inferred substitutions, as indicated by the scale.

A farmed 2-year-old female white-tailed deer exhibited excessive salivation, lethargy, separation from the herd, and excessive recumbency 4 days prior to death on 26 September 2019. At necropsy, the main gross lesions were hepatic congestion and pulmonary congestion/edema. The splenic tissue was processed for virus isolation in C6/36 cells and VeroE6 cells as previously described (5), and cytopathic effects were observed at 7 days postinoculation only in C6/36 cells. Viral RNA was extracted from the clarified supernatant of C6/36 cell culture medium using a QIAamp viral RNA minikit (Qiagen, Valencia, CA) according to the manufacturer’s instructions and served as the template for the construction of a cDNA sequencing library using a NEBNext Ultra II RNA library prep kit (New England Biolabs). The library was sequenced using a v3 chemistry 600-cycle kit on a MiSeq sequencer (Illumina), as previously described (6). A total of 2,635,028 paired-end reads with an average read length of 252 bp were obtained and *de novo* assembled using SPAdes v3.13.0 with default parameters (10). BLASTX searches of the resulting contigs, using OmicsBox v1.2 against the National Center for Biotechnology Information nonredundant protein database, recovered the complete coding sequences for all 10 segments of a Yunnan orbivirus (YUOV) (Table 1). The total length of the complete coding sequences of the 10 YUOV segments was 18,792 bp, with a GC content of 41.3%. BLASTP searches of all 10 proteins (VP1 through VP7 and NS1 through NS3) of the YUOV isolated from a white-tailed deer (OV1288) showed the highest amino acid (aa) identity (97.18 to 99.68%) to YUOV strains. Maximum-likelihood (ML) phylogenetic analyses were performed based on separate amino acid alignments of the outer capsid protein (VP3) and VP2 protein sequences for 41 orbiviruses using IQ-TREE v1.4.4 (11). The best-fit model (LG+F+I+G4) and clade support were determined within IQ-TREE using the Bayesian information criterion and by running 1,000 nonparametric ultrafast bootstraps, respectively. The YUOV isolated from a white-tailed deer (OV1288) was a member of the serotype 1 YUOV clade (Fig. 1) (12).

**TABLE 1:** GenBank accession numbers, genome characteristics, segment descriptions, and top BLASTP hits for the YUOV isolated from a white-tailed deer (OV1288) in Florida

Segment no.	Nucleotide size (bp)	% GC content	Nucleotide accession no.	Protein encoded and structure/function	Data for top BLASTP hit:		
					BLASTP description	Identity (%)	Protein accession no.
1	3,948	39.3	MW424401	RNA-dependent RNA polymerase (VP1)	VP1 (Yunnan orbivirus)	97.18	QGU18499.1
2	2,823	41.6	MW424402	Inner capsid protein (VP2)	VP2 (Middle Point orbivirus)	99.68	ABU95015.1
3	2,622	39.2	MW424403	Outer capsid protein (VP3)	VP3 (Yunnan orbivirus)	97.25	YP_443927.1
4	1,938	41.5	MW424404	Capping enzyme (VP4)	VP4 (Yunnan orbivirus)	99.22	QGU18492.1
6	1,608	43.2	MW424405	Outer capsid protein (VP5)	VP5 (Yunnan orbivirus)	99.25	QGU18502.1
9	1,017	44.4	MW424406	ssRNA and dsRNA binding helicase (VP6)	VP6 (Yunnan orbivirus)	98.52	QGU18494.1
8	1,068	46.5	MW424407	Inner capsid protein (VP7) gene	VP7 (Yunnan orbivirus)	98.87	YP_443932.1
5	1,698	38.7	MW424408	Tubule-forming protein (NS1)	NS1 (Yunnan orbivirus)	98.58	QGU18505.1
7	1,308	43.6	MW424409	Viral inclusion body matrix protein (NS2)	NS2 (Yunnan orbivirus)	99.31	QGU18496.1
10	762	44.9	MW424410	Glycoprotein (NS3)	NS3 (Yunnan orbivirus)	99.21	QGU18497.1

YUOV was first isolated from Culex tritaeniorhynchus mosquitoes collected in Yunnan Province, China (13). Similar to the present study, the Chinese YUOV was isolated in a mosquito cell line (C6/36) but not in mammalian cell lines (13). Two Indonesian YUOVs were isolated from Anopheles vagus mosquitoes in C6/36 cells and Mansonia uniformis mosquitoes in AP-61 cells, but both were refractory to growth in VeroE6 cells (14). Additional YUOVs have been isolated from mosquitoes (A. scapularis) in C6/36 cells and domesticated mammals experiencing neurological disease in Peru, and these same viruses did not grow in mammalian cell lines (15, 16). Phylogenetic analysis of a Middle Point orbivirus isolated from an overtly healthy cow in Australia, along with one of the aforementioned YUOVs isolated from *A. vagus*, identified a second YUOV serotype (12, 17).

Our study confirms that YUOV is present in North America and expands the host range to include white-tailed deer. Future research is needed to better define the mammalian host range of YUOVs and their potential role in disease among wild and farmed mammal populations, including white-tailed deer.

### Data availability.

The genome and raw sequence data for Yunnan orbivirus isolate OV1288 have been deposited in the NCBI GenBank and Sequence Read Archive (SRA) databases under accession no. MW424401 to MW424410 and SRX9773995, respectively.
